# A rare case of iliac crest metastasis from acinic cell carcinoma of parotid gland

**DOI:** 10.1186/1477-7819-12-48

**Published:** 2014-03-01

**Authors:** Sergio Sessa, Antonio Ziranu, Giulio Di Giacomo, Almadori Giovanni, Giulio Maccauro

**Affiliations:** 1 Department of Geriatrics, Orthopedics and Neurosciences, Agostino Gemelli University Hospital, School of Medicine, Catholic University of the Sacred Heart, Rome, Italy; 2 Department of Otolaryngology, Agostino Gemelli University Hospital, School of Medicine, Catholic University of the Sacred Heart, Rome, Italy

**Keywords:** Parotid gland, Pelvic metastasis, Acinic cell carcinoma

## Abstract

A case of acinic cell carcinoma of the right parotid gland metastasizing to the right iliac crest is presented. Generally, for this rare low-grade malignant salivary gland neoplasm, 20% of cases may have local recurrences whereas about 10% of cases have distant metastases. They may arise many years after the initial presentation of the original tumor. The most frequent locations are the cervical lymph nodes, liver, lungs, contralateral orbit and bones. Occurrence in the appendicular skeleton is very rare and in our knowledge this is the first report of metastases to the pelvis.

## Background

There are many histological types of salivary gland neoplasms. They include benign and malignant tumors of epithelial, mesenchymal and lymphoid origin. The 2005 World Health Organization (WHO) Classification of salivary gland tumors is complex and includes 10 benign and 23 malignant entities of epithelial origin [1]. Non-epithelial neoplasms are rare.

Of the salivary glands, the parotid is the most frequently affected by neoplasms (accounting for 75% of the total) [2]. Generally these lesions are benign and only 25% of them are malignant. Acinic cell carcinoma is an uncommon malignancy of salivary gland origin. The parotid gland is the most common primary site. Interestingly, this type of carcinoma was considered benign until 1953 when Buxton demonstrated its ability to metastasize and recur locally [3]. Moreover it has been observed that an acinic cell carcinoma can recur many years after the primary diagnosis and subsequent removal [4]. Spiro reported two cases of recurrence after 30 years of follow-up [5]. However, many authors has asserted that 20 years of follow-up is adequate [6,7]. The percentage of distant metastases from salivary gland tumors is relatively low and their occurrence is associated with high-grade tumors [8]. Metastases occur in bones and the axial skeleton is the most common site. We report the case of a 37-year-old man with an acinic cell carcinoma of a parotid gland metastasizing to the iliac crest 3 years after resection of the primary tumor.

## Case presentation

**Figure 1:**
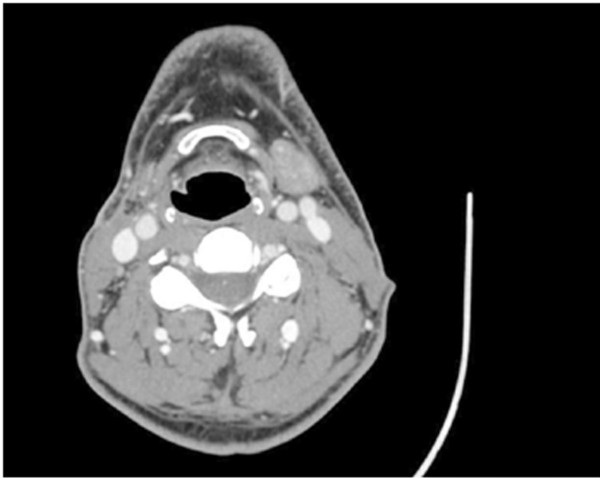
Axial CT scan showing the involvement of the right parotid region.

**Figure 2:**
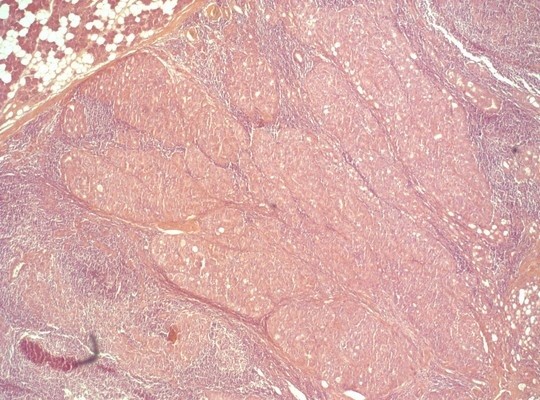
Histologic section showing the solid growth pattern of the neoplasm.

In June 2008, a man went to our hospital because of a harsh, fixed and indolent swelling in the parotid region. He had an ultrasound exam, which showed three swollen lymph nodes (maximum diameter 2.1 cm) near the parotid gland. Computed tomography (CT), magnetic resonance imaging (MRI) and a positron emission tomography–computed tomography (PET-CT) scan confirmed there was a tumor in the right parotid region with ipsilateral lymph node involvement (Figure 1). A cytological examination by needle aspiration revealed carcinoma cells. In September 2008, the patient underwent a right parotidectomy without resection of the facial nerve and emptying of the right lateral cervical metastases. After a histological examination, he was diagnosed with a dedifferentiated acinic cell carcinoma with a solid growth pattern (Figure 2). Subsequently he received 3 months of adjuvant radiotherapy.

In May 2011, the man was readmitted to our hospital because of right-sided Bell's palsy. MRI, a PET-CT scan and needle aspiration showed and confirmed the recurrence of a poorly differentiated carcinoma (dedifferentiated acinic cell carcinoma). Between May and September 2011, the patient underwent a right parotidectomy revision together with bilateral laterocervical emptying. Subsequently catheters for brachytherapy were positioned (total dose of 3,000 cGy) and he received systemic chemotherapy with cisplatin and 5-fluorouracil.

**Figure 3:**
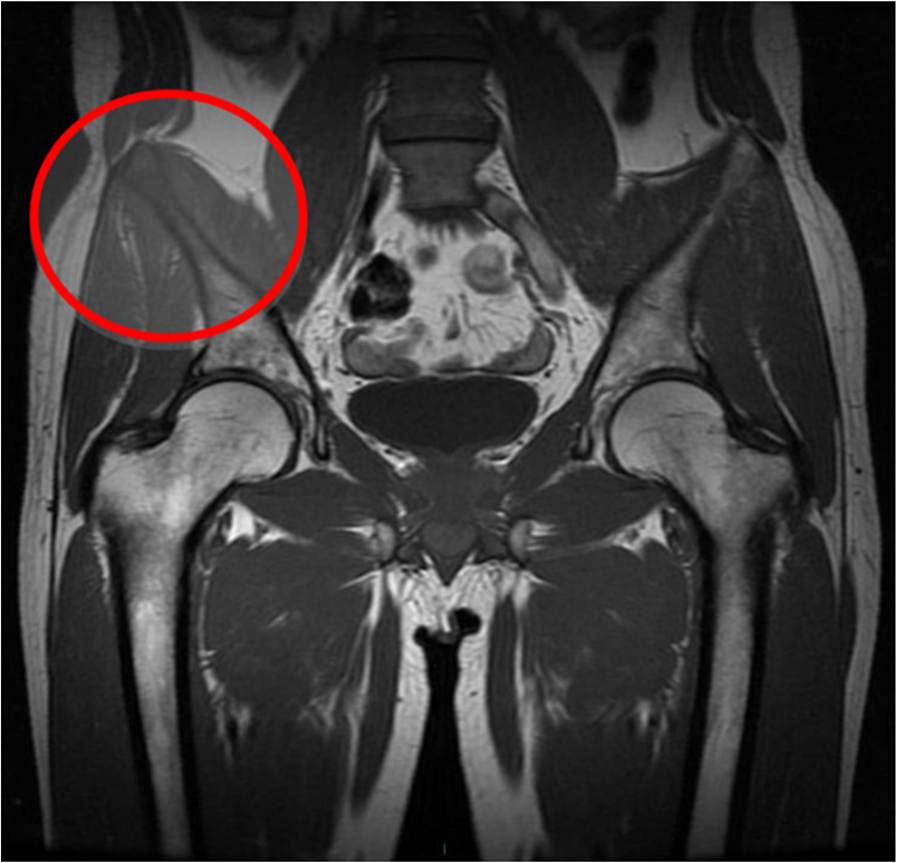
Coronal MRI showing the involvement of the right iliac crest.

**Figure 4:**
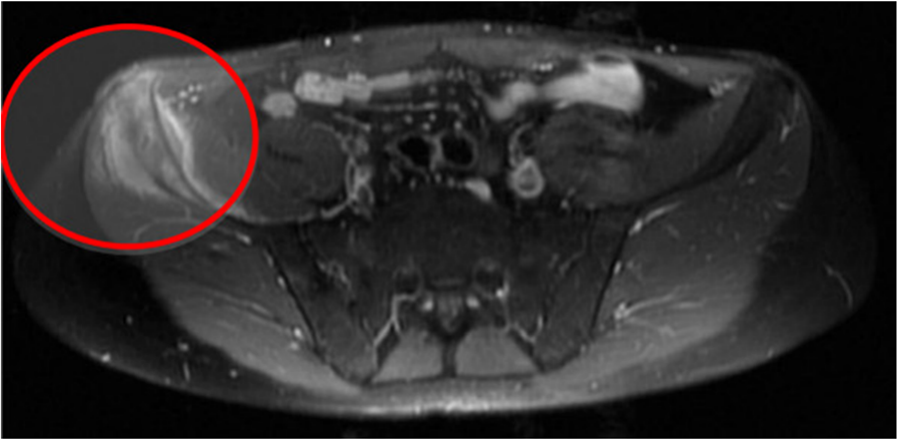
Axial MRI showing the involvement of the right iliac crest.

**Figure 5:**
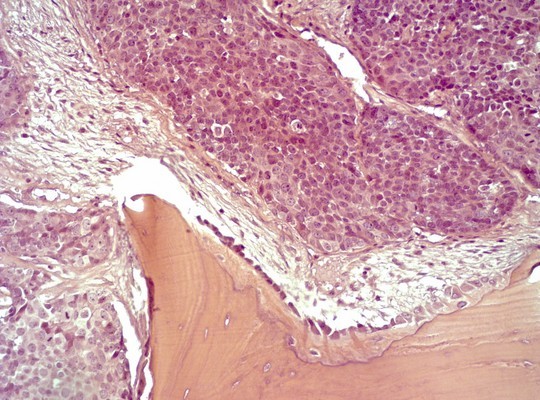
Histologic section showing infiltration of bone by the tumor.

In October 2011, the patient presented to our observation because of an acute pain in the right hemipelvis. He had an MRI scan, which showed an area of morphostructural alteration, hypointense on T1 sequences and hyperintense on T2 sequences, in the right iliac crest. The area was surrounded by solid tissue that had permeated the anterior portion of the iliac crest and extended into the endo- and extrapelvic soft tissues (iliacus and gluteus minimus muscles) (Figures 3, 4 and 5). There were no transformations between the primary parotid tumor and the metastatic lesions. The solid tissue extended, inferiorly, into the bone marrow up to 6 cm from the iliac crest. The patient underwent a surgical resection of the right iliac crest and reconstruction with acrylic cement.

### Surgical technique

**Figure 6:**
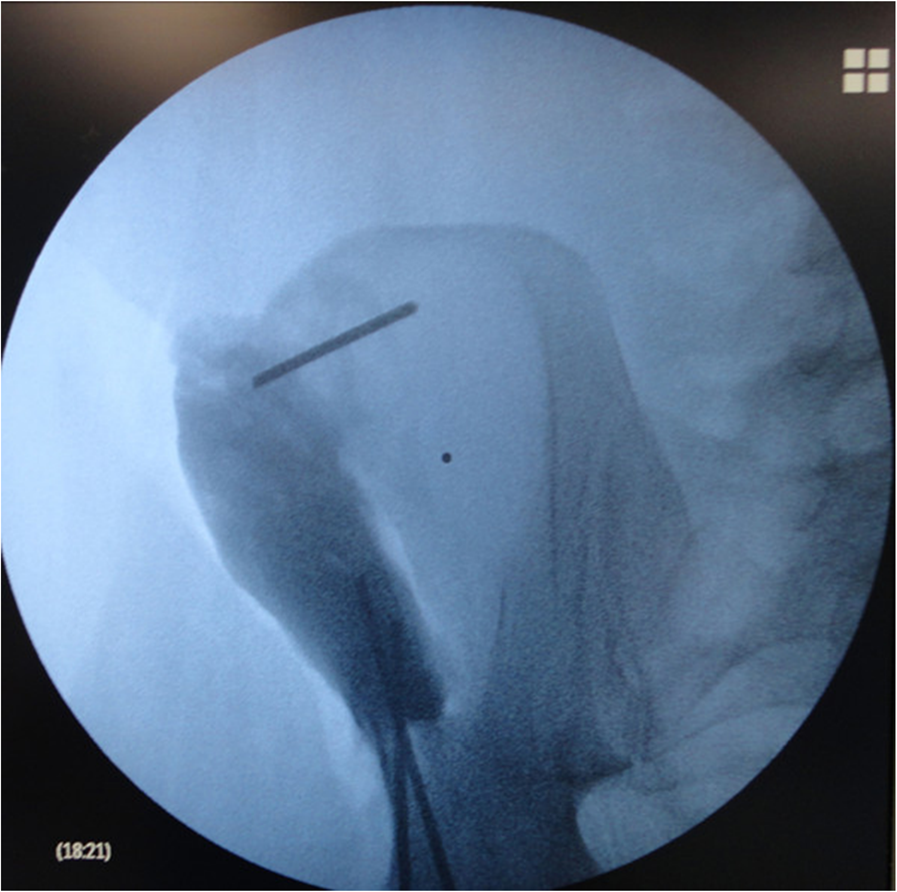
Intraoperative X-ray showing TEN insertion in the iliac crest.

**Figure 7:**
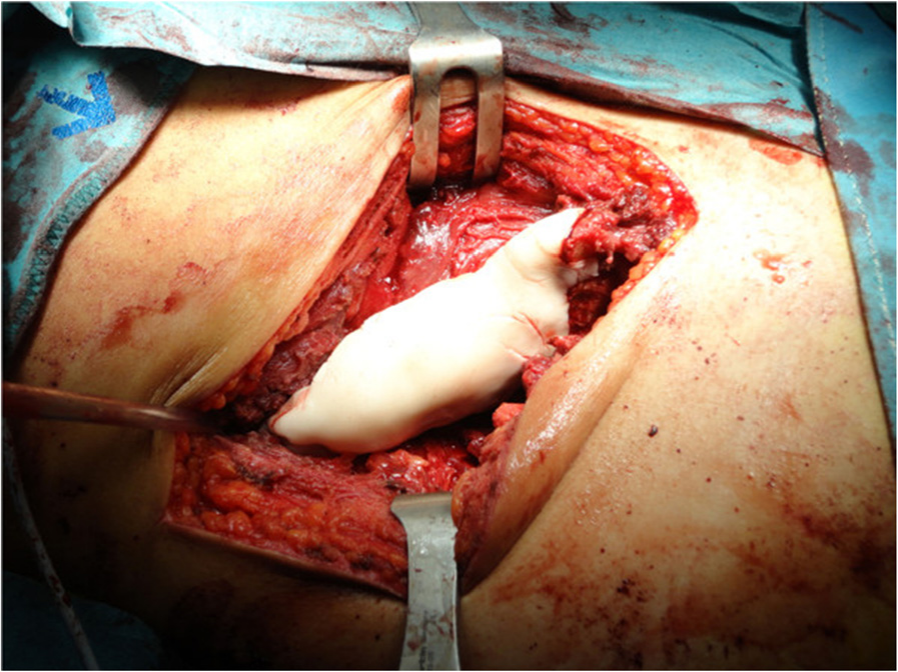
Acrylic cement reconstruction of the iliac crest.

**Figure 8:**
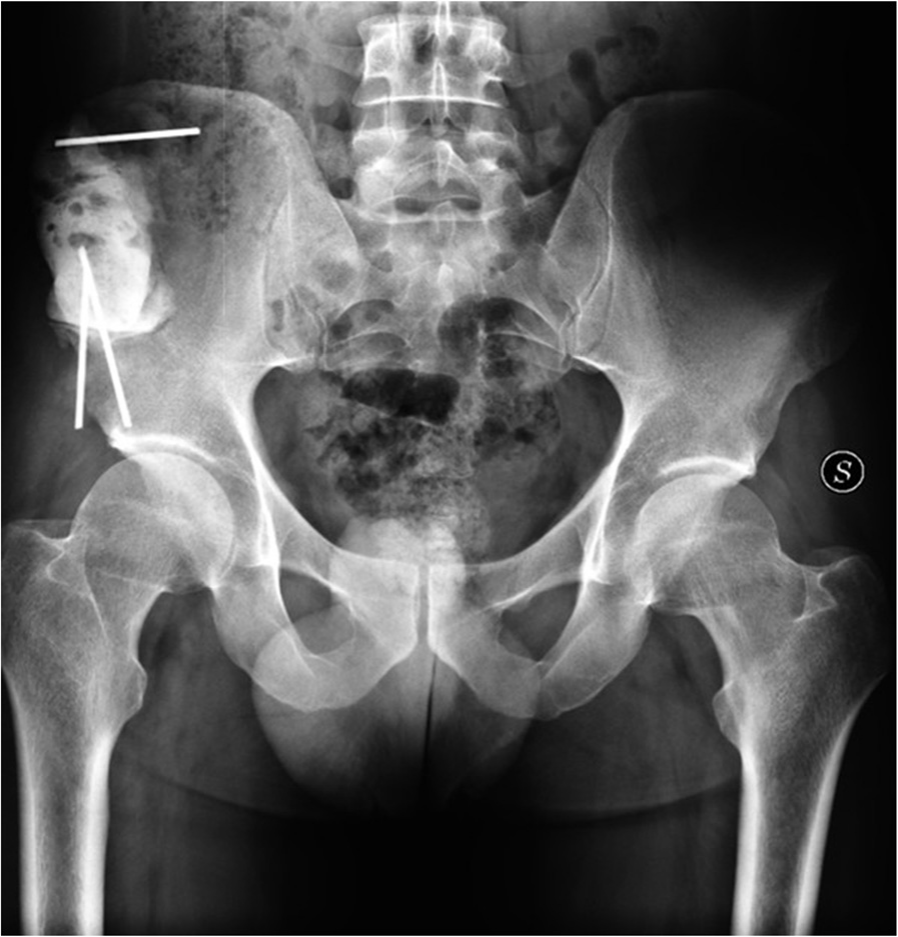
One-year post-operative X-ray.

The patient was placed in the supine position and an ilioinguinal approach according to Letournel was performed [9]. The main incision began at the posterior portion of the iliac crest, then curved forward along the course of the iliac crest as far as the anterior superior iliac spine and ended about two fingerbreadths from the cranial side of the symphysis. This approach gives good exposure of the retroperitoneal space as well as the posterior retrogluteal area and permits a safe resection of the ilium. After transection of the skin and subcutis, the iliac crest was fully exposed subperiosteally. We observed a neoplastic contamination of the muscular tissues that surround the iliac crest. All of these tissues were excised and histologically analyzed. Then we proceeded with the resection of the infiltrated iliac crest (Type I pelvic resection according to the Enneking and Dunham classification [10]). The tissue resected was histologically analyzed. The iliac crest was reconstructed by pinning with two titanium wires (TEN) 3.0 mm in diameter in the distal margin and one in the proximal margin of the resection zone and using acrylic cement (Figures 6 and 7). The final histological report showed that the tissue from the salivary gland was neoplastic (dedifferentiated acinic cell carcinoma). The patient received adjuvant chemotherapy after surgery. A pelvic radiograph after 1 year showed there was good positioning of the metal wires and cement (Figure 8).

### Discussion

Acinic cell carcinomas are a type of uncommon salivary gland tumor, which were considered benign until 1953 when Buxton demonstrated their ability to metastasize and to recur locally [3]. Subsequently they were referred to as acinic cell carcinomas. They are considered, however, to be low-grade malignant tumors. This type of tumor recurs locally in 8.3% to 45% of cases and these recurrences may arise many years after the original diagnosis of cancer. In the literature there are reports of cases of local recurrence 30 years after the diagnosis of the primitive tumor [5]. As with other primary malignant parotid tumors, they rarely cause distant skeletal metastases [11]. They are found more frequently in the axial skeleton and especially in the thoracic spine [12-14]. A new tumor entity of the salivary glands, mammary analogue secretory carcinoma (MASC) with *ETV6*-*NTRK3* translocation, has recently been proposed. MASCs are classified as adenocarcinomas, not otherwise specified (ANOS) or acinic cell carcinomas (AciCC) by the current World Health Organization classification. MASCs can be differentiated from acinic cell carcinomas by a lack of periodic acid-Schiff diastase–positive zymogen granules and S-100 protein positivity. Immunohistochemically, the lesional cells of our patient were negative for S-100 protein and they contained Periodic Acid Schiff-positive secretory zymogen granules: hence the patient’s tumor was an acinic cell carcinoma.

The peculiarity of our case was the presence of a bone metastasis, originating from an acinic cell carcinoma of a parotid gland. It was in the right iliac crest in the appendicular skeleton. The pelvis is the second most common site of bone metastases after the spine, for this type of salivary gland tumor. Generally, this type of bone lesion shows through the onset of pain, mechanical instability due to extensive bone destruction and pathological fractures. For this reason, the goals in the treatment of these lesions are the control of pain, the prevention and treatment of fractures, maintaining the patient's independence and preventing progression of the tumor. Surgeons, radiotherapists, medical oncologists and pain clinicians should work together to improve the longevity and the quality of life of patients.

Lesions that do not involve the hip joint, such as those in the ischium, pubis or sacroiliac area, generally may be treated non-operatively [15,16] with radiation alone or using minimally invasive procedures such as radiofrequency ablation, cryosurgery and/or percutaneous cementoplasty, following the technique proposed for acetabular metastasis [17].

The authors decided to reconstruct the iliac crest to avoid leaving a significant defect in the pelvic bone, which could cause pain at the donor site, instability of the pelvis [18], fractures of the ilium [19], donor site muscle herniation or abdominal content herniation [20]. Surgical indications for pelvic bone metastases are given by the Capanna and Campanacci Classification [21]. For our patient, the presence of a solitary metastasis, a primitive tumor with good prognosis (acinic cell carcinoma), 3 years since detection of the primary tumor (Class 1 of the Capanna and Campanacci Classification) and considering the specific clinical case (young patient, pain resistant to medical treatment), the surgical technique chosen was a resection with wide surgical margins, following the Enneking and Dunham Classification (P1), with reconstruction using acrylic cement and TEN.

## Conclusions

This case report of an acinic cell carcinoma in a parotid gland with histologically confirmed solitary metastasis to the iliac crest treated by surgical resection and reconstruction with acrylic cement and TEN is a very rare case in the literature. To our knowledge, this is the first reported case of an acinic cell carcinoma in a parotid gland metastasizing to the iliac crest in an adult.

## Consent

Written informed consent was obtained from the patient for publication of this case report and accompanying images. A copy of the written consent is available for review by the Editor-in-Chief of this journal.

## Abbreviations

CT: computed tomography; MASC: mammary analogue secretory carcinoma; MRI: magnetic resonance imaging; PET-CT: positron emission tomography–computed tomography.

## Competing interests

We declare no competing interests for all authors.

## Authors’ contributions

SS wrote the manuscript. SS, GM and GA performed surgery. SS, AZ and GDG were involved in the final editing. All authors approved the final manuscript.
